# Clinical Implementational and Site-Specific Workflows for a 1.5T MR-Linac

**DOI:** 10.3390/jcm11061662

**Published:** 2022-03-16

**Authors:** David A. P. Dunkerley, Daniel E. Hyer, Jeffrey E. Snyder, Joël J. St-Aubin, Carryn M. Anderson, Joseph M. Caster, Mark C. Smith, John M. Buatti, Sridhar Yaddanapudi

**Affiliations:** Department of Radiation Oncology, University of Iowa Health Care, Iowa City, IA 52242, USA; david-dunkerley@uiowa.edu (D.A.P.D.); daniel-hyer@uiowa.edu (D.E.H.); jeffrey-snyder@uiowa.edu (J.E.S.); joel-st-aubin@uiowa.edu (J.J.S.-A.); carryn-anderson@uiowa.edu (C.M.A.); joseph-caster@uiowa.edu (J.M.C.); mark-c-smith@uiowa.edu (M.C.S.); john-buatti@uiowa.edu (J.M.B.)

**Keywords:** adaptive radiotherapy, MRI, MRgART, treatment planning, Elekta Unity

## Abstract

MR-guided adaptive radiotherapy (MRgART) provides opportunities to benefit patients through enhanced use of advanced imaging during treatment for many patients with various cancer treatment sites. This novel technology presents many new challenges which vary based on anatomic treatment location, technique, and potential changes of both tumor and normal tissue during treatment. When introducing new treatment sites, considerations regarding appropriate patient selection, treatment planning, immobilization, and plan-adaption criteria must be thoroughly explored to ensure adequate treatments are performed. This paper presents an institution’s experience in developing a MRgART program for a 1.5T MR-linac for the first 234 patients. The paper suggests practical treatment workflows and considerations for treating with MRgART at different anatomical sites, including imaging guidelines, patient immobilization, adaptive workflows, and utilization of bolus.

## 1. Introduction

After over a decade of development, the integration of magnetic resonance (MR) imaging and linear accelerators has been realized within the context of clinical radiation therapy [1,2,3,4,5,6]. The excellent soft-tissue contrast and ability to track targets in near-real time without additional ionizing radiation are key distinguishing features that are driving the widespread adoption of MRI-guided linear accelerators. The inclusion of daily MRI provides an opportunity for quantitative imaging throughout the treatment. In addition to the hardware developments inherent in the MR-linacs, software systems have also been developed congruently that allow for MR-guided adaptive radiotherapy (MRgART) treatments with the patient on the treatment table [3]. The imaging and adaptive capabilities provide multiple potential benefits for radiation treatments such as permitting isotoxic dose escalation or organ-at-risk (OAR) sparing objectives, facilitating the reduction of treatment margins, improved confidence treating near critical structures, and improving dose-tracking metrics. While these combined hardware and software systems have the potential to improve treatment accuracy, delivering treatments in the presence of a magnetic field and performing online plan adaptions present significant challenges that must be considered [7]. The functionality provided by MRI-guided linear accelerators requires a thorough understanding of system limitations which have variable impact on individual patients and the location of the treated area. This work explores one institution’s experience and approach in navigating practical clinical implementation, including treatment planning and delivery to various treatment sites during the development of an MRgART program. The workflows presented are specific to a single institution but can provide a useful foundation for any center building a new program through the presentation of case studies and discussion of clinical rationale. It is not the purpose of this work to discuss commissioning of a MRgART program; for that discussion, readers are referred to other works [8,9,10,11,12,13].

## 2. Materials and Methods

### 2.1. MR-Linac

The Elekta Unity MR-linac (Elekta Unity, Elekta AB, Stockholm, Sweden) used in this body of work consists of a Philips 1.5T MR imaging system (Philips Healthcare, Amsterdam, the Netherlands) coupled with a single-energy linear accelerator capable of producing a 7-megavolt (MV) flattening filter-free (FFF) photon beam with a source-axis distance (SAD) of 143.5 cm and a nominal dose rate of 425 MU/min at isocenter [1]. Treatments are delivered using step-and-shoot intensity-modulated radiation therapy (IMRT) and patients are positioned within the 70 cm bore of the MRI. A multileaf collimator system, similar to the Agility multileaf collimator (MLC) on Elekta Versa HD linear accelerators, includes 160 leaves capable of shaping fields for step-and-shoot IMRT with a maximum field size of 57.4 × 22 cm [8].

The 1.5T MRI can perform imaging prior to treatment for registration and setup, during treatment planning or treatment, and after treatment is complete. Additionally, for all sites excluding brain, live motion-monitoring cine imaging of the cardinal planes is available at 5 frames per second (FPS).

This MR-linac is capable of performing adaptive radiotherapy using two primary workflows: adapt-to-position (ATP) and adapt-to-shape (ATS). The ATP workflow is a standard image-guided radiotherapy (IGRT-based) workflow and is able to be quickly executed but restricted to a rigid translational registration and a limited optimizer that works to strictly reproduce dose-volume histogram (DVH) characteristics of the reference plan, while the latter (ATS) permits recontouring and access to the full treatment-planning system optimizer.

### 2.2. Clinical Setting

The adaptive radiotherapy program is located at an academic medical center in the United States that includes 8 subspecialized radiation oncologists. The MRgART program is overseen by 5 medical physicists and planning is performed by 2 dosimetrists with adaptive-radiotherapy-specific Monaco training. Six radiation therapists, dually certified in radiation therapy and MRI, perform patient screening prior to MR simulation, setup, and delivery to ensure MR safety procedures are followed and operate the MRI portion of treatment. They also perform an integral role during the MRI portion of pretreatment patient simulation, including performing navigator-triggered imaging and patient compression as a method of motion management. The therapists ensure that vital information is communicated between the physicists and attending radiation oncologists between treatments, as their schedule provides a consistent presence throughout a treatment course. The facility has staffed a 3T MR simulator since 2005 and has a history of dual-certified staff for MR-based procedures.

### 2.3. Patient Selection

Patients were selected based on physician perception that they would benefit from the improved soft-tissue contrast, motion monitoring, daily adaption, or any combination of the three. Common contraindications for treatment on the 1.5T MR-linac included the presence of ferromagnetic implants or non-MR-compatible devices, severe claustrophobia, restrictively large body habitus exceeding the receiver coil bridge or bore size, or inability to hold the immobilized position for the entire treatment duration. Patients whose treatment target exceeded the field size limits of the 1.5T MR-linac system were also excluded.

### 2.4. Patient Population

**Figure 1:**
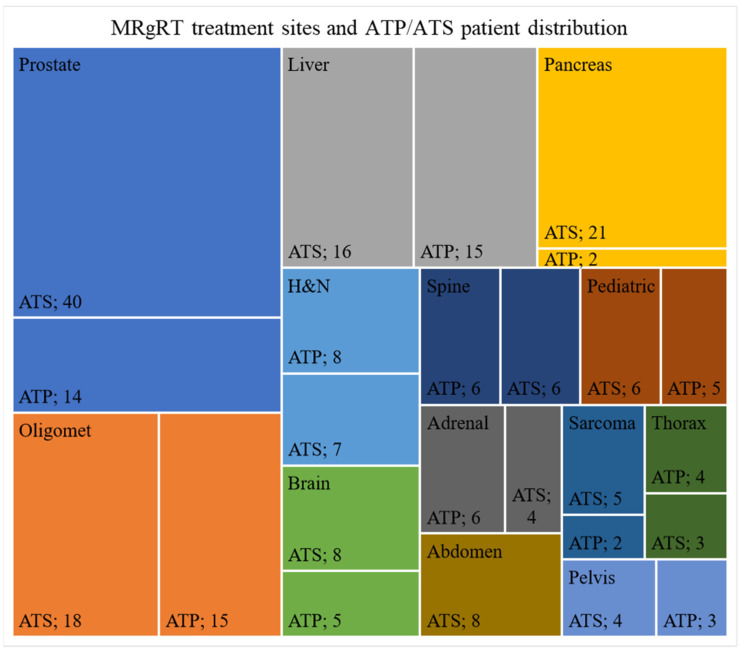
Distribution of treatment sites and patients treated with at least one ATS fraction or all ATP treatments.

From 22 May 2019 to 1 December 2021, 234 patients received treatment using MRgRT, and of these patients 121 were treated using a stereotactic body radiotherapy (SBRT) technique. For these patients, 3242 fractions were delivered with 594 (18.3%) following the adapt-to-shape (ATS) workflow and 2648 (81.6%) with the adapt-to-position (ATP) workflow. The most commonly treated site was prostate (23%) with oligomets (14%), liver (13%), and pancreas (10%) being the next most common sites of treatment. The total distribution of treatment sites and patients treated with ATP or with at least one ATS fraction can be found in Figure 1.

### 2.5. General Adaptive Radiotherapy Workflows

**Figure 2:**
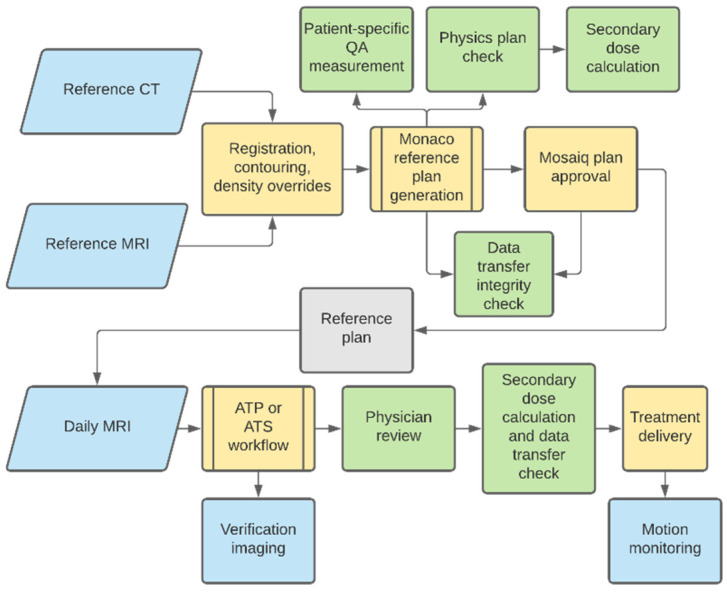
Workflow diagram showing major processes in treatment-plan generation and delivery. Imaging steps noted in blue, quality assurance (QA) steps noted in green, and planning or delivery steps noted in yellow.

The planning tools provided by the Monaco treatment-planning system in conjunction with the daily MRI-guided adaptive treatment planning. Briefly, pretreatment planning (initial simulation-based treatment planning), referred to as reference planning, is identical to traditional radiation-treatment-planning workflows (detailed in the following section). On the day of treatment delivery, a new MRI is obtained and a decision is made while evaluating the daily MR image registration to select the adapt-to-position or adapt-to-shape MRgART workflows (Figure 2). In our institution’s experience, the time required for daily ATP treatment planning was 14 min and ATS treatment planning was 25 min and improved with user experience [14].

### 2.6. Pretreatment Workflow

**Figure 3:**
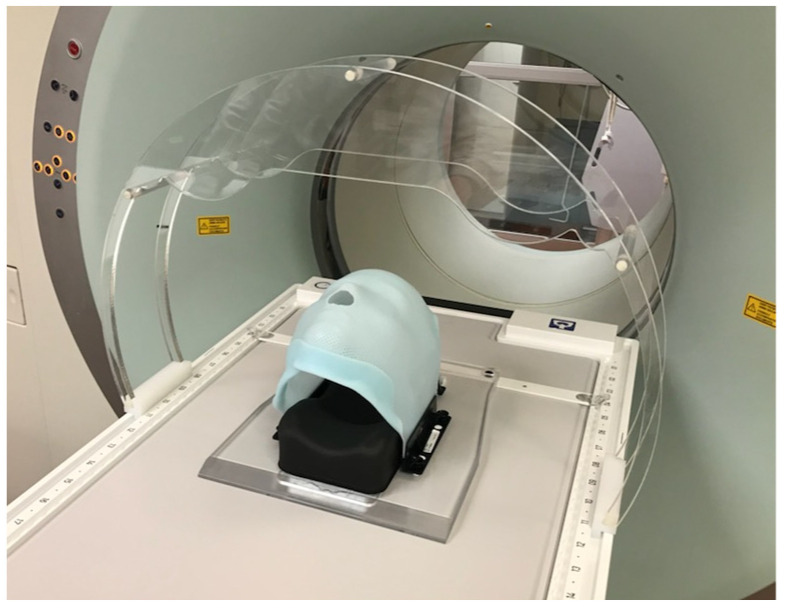
Acrylic replica of anterior coil bridge to test for patient clearance during simulation.

Prior to the first treatment, an initial reference treatment plan based on simulation imaging is created for each patient. Patient simulation consists of both CT (or CT/positron emission tomography (PET)) and MRI components with MRIs acquired on a 3T Siemens Magnetom Trio (Siemens, Munich, Germany) and CTs or CT/PET acquired with a Siemens Biograph 40 PET/CT scanner. MRIs are acquired using multiple sequences that aid in OAR and target delineation. The simulation MRI sequences can also be used to determine the scan protocol best suited for registration during daily imaging. For MRI simulation, T1 and T2 scans are acquired, with additional scans acquired based on treatment location, use of contrast, or per the attending physician’s request. During this imaging, the patient is observed for anxiety and comfort in the immobilization devices that may rarely contraindicate daily treatment on a closed-bore system. Due to patients not being simulated on the MR-linac, which provides more treatment time on the machine, clearance for the imaging coil bridge is also tested using an in-house-developed acrylic replica of the MR-linac anterior coil to ensure that there will be adequate clearance during treatment (Figure 3). As one of the early adopters of the 1.5T MR-Linac, our institution developed our own clearance tool for use during treatment simulation, but Elekta now provides a similar tool as part of the purchase.

Treatment planning is performed using the Monaco treatment-planning system V5.51.10 by dosimetrists trained in MRgRT planning. The MRI, PET, and CT images are registered, and the fusion used for target and OAR delineation. Information for the bulk electron-density overrides used for adapt-to-shape (ATS) planning are extracted from the contoured CT structures, taking into consideration the use of contrast or artifacts which may distort the electron-density information. The layering order of the electron-density assignments are of significant importance, as these values will automatically propagate to daily MRI images during all ATS workflows. In general, in our clinical practice, organs with contrast are overridden to electron-density values derived from the average of a similar cohort of patients.

Templates with arrangements of 9 to 11 beams are commonly used for plan generation. Each beam arrangement is selected to avoid the high-density couch angles located approximately between 110°–135° and 230°–255° and the cryostat pipe at 13° [8]. The initial beam orientations are adjusted as required to avoid OARs and high-density implants.

Once an acceptable plan is created, a secondary dose calculation is performed using a version of RadCalc V6.3 (Lifeline Software Inc., Tyler, TX, USA) modified to model the unique system geometry. A data-transfer integrity check, comparing the plan parameters between the treatment-planning system (TPS) and Mosaiq record and verify system, is also completed using RadCalc [9]. Although this version of RadCalc utilizes a modified Clarkson integration, which does not model the magnetic field, a reasonable agreement was achieved between the model and the TPS, ultimately allowing us to implement a 3% tolerance level and 5% action level for calculations. All reference plans were measured using a Sun Nuclear MR-compatible ArcCheck (Melbourne, FL, USA) using TG-218 3%/2mm gamma-criteria recommendations [15].

### 2.7. Online Workflow

The pretreatment daily MRI is registered to the image set used for planning, most commonly a CT scan at our institution, and evaluated by the medical physicist to determine whether the ATP or ATS workflow is needed. Common parameters evaluated include patient alignment to the reference images, changes in tumor volume, image quality of MRI, and organ location and size. Factors influencing the decision can include the use of larger planning target volume (PTV) and planning organ-at-risk volume (PRV) margins which are used when intrafraction motion or organ expansion is expected. If patient anatomy sufficiently agrees with the simulation scans, the ATP workflow is performed. Of the available optimization options, only the optimize-weights-and-shapes method is used due to the improved plan quality relative to the faster optimization options [3]. The final registration and plan are evaluated by the physician. During the registration process, it is important to evaluate not only the alignment of the target but also of nearby organs at risk, as the ATP optimization aims to reproduce the reference plan DVH. Organs at risk that do not align well with the reference plan will lead to inaccurately reported DVH values. Thus, a close inspection of the isodose lines relative to the underlying MRI anatomy is also performed by the physician and medical physicist.

When patient anatomy significantly varies relative to the reference plan or a dosimetric objective is difficult to achieve with the ATP optimization method, the ATS workflow is used. The institutional workflow for ATS involves recontouring of the targets by the attending physician and verifying OAR contours that have been recontoured or deformed by the physicist to the current patient anatomy. The plan is then generated, and bulk density overrides are verified. Due to the longer time required for the ATS workflow, a verification MRI is acquired during the optimization phase to ensure the patient has not moved and organs remain in a similar location [2]. At this institution, it is estimated that between 1% and 3% of ATS fractions are replanned due to anatomic or patient positioning changes that occur during recontouring and planning. To mitigate the number of replanning at this stage, a 2–3 mm margin may be added to highly mobile or distensible organs (e.g., small bowel) to account for the intrafraction motion during the ATS workflow. The verification image alignment and final plan are evaluated by the physician. If the patient did move between the initial daily MRI image and the conclusion of ATS optimization, an ATP workflow will be performed using the daily ATS plan as a reference. This final ATP plan will be used for treatment delivery. A record of the changes performed for each ATS optimization is kept in order to develop a library of plans for each patient, allowing for a quick reference to plans that have a higher probability of matching the daily anatomical changes and perform ATP from one of these library plans in future fractions.

For both ATP and ATS treatment options, data-transfer integrity and secondary dosimetric calculations are performed using RadCalc for each fraction [9]. Additionally, ArcCheck measurements were performed for the first 512 fractions until a statistical evaluation was performed demonstrating confidence that adaptive plans are expected to be acceptable if the reference-plan patient-specific QA measurement was also acceptable [10].

## 3. Results

### 3.1. Liver with Motion and with Compression Applied

Targets and OARs located in the abdomen can benefit from MRgRT due to the excellent soft-tissue contrast to distinguish the organs clearly and the ability to adapt target and OAR volumes daily. However, targets and OARs in the abdomen may be highly mobile with daily variations in position, while targets located near the diaphragm (liver, pancreas, etc.) may also be influenced by respiratory motion [16].

**Figure 4:**
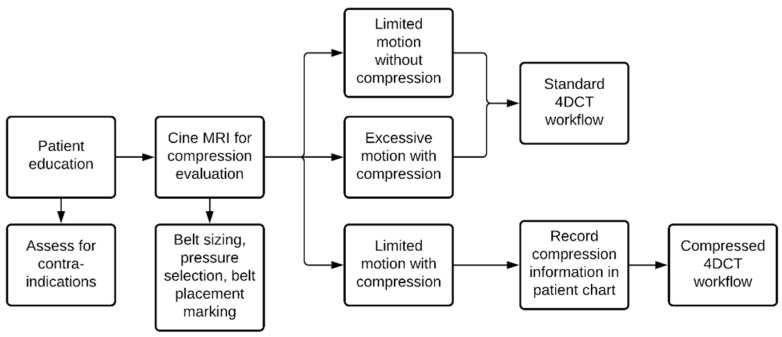
Compressed patient simulation workflow.

As seen in Figure 1, liver cancer treatments make up a significant fraction of the patient population treated using MRgRT at our institution. To account for respiratory motion during planning, an internal target volume (ITV)-planning strategy with the possibility of abdominal compression is used pending the release of respiratory-based gating. An overview of the simulation decision workflow is shown in Figure 4. Briefly, a patient is educated prior to simulation and screened for contraindications of abdominal compression such as hernias or ostomy bags. The patient is then taken to the MRI simulator and a cine MRI is used to determine the effectiveness of the abdominal compression on an individual-patient basis. A sagittal and coronal cine MRI (TE: 3.8 ms, TR: 1.92 ms, flip angle: 40 deg) are acquired over 60 s to capture multiple breathing cycles. First, a cine MRI with no abdominal compression is acquired where the motion of the diaphragm in the superior/inferior and anterior/posterior directions are measured as a surrogate for the liver tumor [17]. If the magnitude of this motion is clinically unacceptable (>15 mm Euclidian), abdominal compression is applied, and a cine MRI is again acquired to reassess the motion magnitude of the diaphragm. If the Euclidian motion is still >15 mm with compression, a physician and physicist consultation is required to determine how to proceed. If it is determined that the motion is too large, the patient may be transferred to a machine with gating capabilities for treatment. If an acceptable amount of motion (<15 mm) is achieved with compression, the compression parameters are recorded, and the MRI-simulation images are acquired with the patient under compression. The tumor motion is determined based on the post-gadolinium-injection cine-MRI images, and this motion is compared to the diaphragm surrogate motion and recorded. The patient then is transferred for computed tomography (CT) simulation. At CT, the patient is compressed as they were during MRI simulation from their recorded chart notes, and an exhale breath-hold scan is acquired. Following this, a four-dimensional computed tomography (4DCT) or inhalation breath-hold scan will also be acquired (if a high-quality 4DCT is unable to be acquired due to patient compliance). It should be noted that this imaging workflow is the same for noncompressed patients with minimal motion (<10 mm), just without the addition of the compression device at MRI or CT.

**Figure 5:**
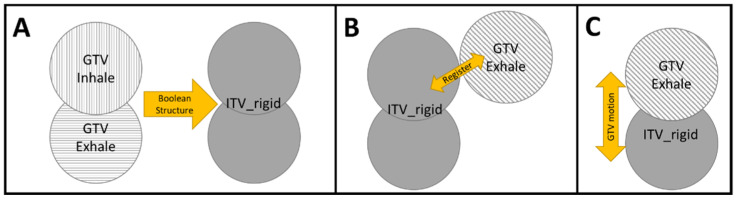
ITV planning and treatment strategy for compression patients. (**A**) An ITV is created from inhale and exhale simulation scans and set as a rigid structure. (**B**) The rigid structure can be registered to the GTV from the navigator-triggered image along the superior edge. (**C**) GTV motion can be verified using motion monitoring.

During planning, the exhale breath-hold scan is used as the primary dataset and the 4DCT (or inhalation CT) scans are used to create an ITV using Boolean operations. This margin structure is then converted to a rigid structure to preserve the motion information which will not be available during the pretreatment navigator-triggered MRI and can be used for PTV generation. Currently, 4D MRI sequences are not part of the standard imaging sequences provided by the vendors. When abdominal compression is used, a pretreatment cine MRI is acquired, and the superior/inferior diaphragm motion is assessed and compared to the motion recorded on the day of simulation. If the diaphragm-motion difference between treatment and simulation is >5mm, the physicist is paged, and additional compression may be applied. Once the diaphragm-motion difference is ≤5 mm, a standard abdominal treatment workflow is used, starting with the acquisition of a navigator-triggered MRI scan which acquires the MRI only at the end expiration-breathing phase [8,18]. The expiration acquisition allows for a proper registration with the exhale breath-hold CT scan that was used for planning. The target, visible on the navigator-triggered MRI, is aligned to the superior edge of the ITV (Figure 5) to ensure that the tumor motion is captured within the ITV. During registration, organ position is evaluated and target movement is evaluated using motion monitoring if necessary. Although most targets located in the liver are visible under the pretreatment MRI, they are likely not visible during motion monitoring. Evaluation of alignment is then performed by referring to nearby and visible anatomic landmarks.

This clinic has delivered 93 fractions to 19 patients using this abdominal compression protocol. Superior/inferior motion of the diaphragm was reduced by 10.6 ± 4.6 mm using abdominal compression for these patients. During treatment, motion was evaluated using a cine MRI during treatment to verify similar conditions to simulation. The average difference in the diaphragm motion between simulation and treatment was 1.3 ± 1.6 mm with a maximum of 6 mm across all fractions.

### 3.2. Head and Neck

**Figure 6:**
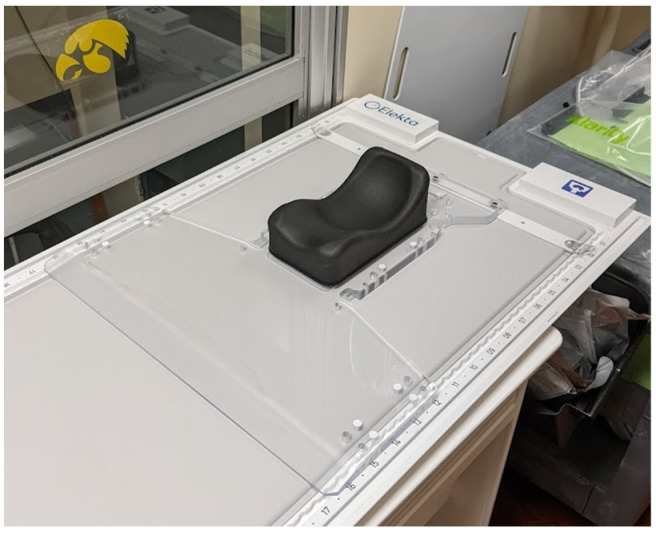
Custom acrylic baseplate for head-and-shoulder mask fixation and indexing.

Patients with head and neck treatments may benefit from adaptive radiotherapy for multiple reasons. The ability to easily visualize and recontour critical organs and targets on MRI allow for precise contouring, alignment, and review of the dose distribution. While immobilization for these treatments includes a head and shoulder mask and a custom acrylic baseplate (Figure 6), flexion of the spine and movement of the mandible and other nonrigid changes in patient anatomy can and do occur. Using adaptive radiotherapy, these deformations can be accounted for by recontouring and optimizing on daily treatments. Finally, these patients are likely to experience weight loss and possible variations in tumor size over the course of treatment and may benefit from MRgART due to response-based changes. In the experience of our institution, it is common for the tumor regression over the course of treatment to allow the reduction of treatment volumes in effort to mitigate secondary toxicities and adapt OARs as the patient loses weight. If the patient’s body habitus changes so much that the head and shoulder immobilization is no longer effective, a complete patient-simulation workflow with a new mask may be performed. Patients are selected for MRgART over conventional fractionation based on the tumor location, potential for tumor regression based on clinical pathology, and proximity to OARs that may be spared dose from frequent replanning which reduces PTV volume.

Additional considerations of the acrylic baseplate must be considered during treatment planning due to the baseplate not being visible. Currently, the Monaco treatment planning system only allows for a single couch structure, so a unique workflow has been developed to ensure proper placement of the treatment couch with respect to the acrylic baseplate and patient external surface. During generation of the reference treatment plan, the acrylic baseplate and patient contour are separately contoured and then combined as a margin structure to create the external contour. Creating the external surface as a margin structure allows the patient contour to be deformed to changing patient anatomy while including the electron density of the acrylic baseplate in the dose calculation. Failure to complete this workflow will result in the couch top being placed such that it overlaps with the acrylic baseplate, which is not representative of the actual treatment setup.

The clinical workflow at our institution includes the use of offline adaptive plans for head and neck cases. Current time constraints for online adaptive radiotherapy for head and neck treatments due to the extensive recontouring can make online adaptions difficult [19]. For patients that have a significant change in target size and other clinically relevant changes in anatomy, the plan will be adapted offline. This workflow permits additional time to provide higher-quality contours than those generated under the time constraints with the patient on the table and allow more patients to be scheduled during the day. This consideration is especially important towards the end of head and neck-treatment courses when radiation-induced side effects may limit the time a patient can comfortably remain in the immobilization device. Additionally, the offline adaptive radiotherapy tools provide the ability to rapidly recontour and replan and utilize far fewer resources and time relative to a traditional radiation therapy approach, which requires a full resimulation of the patient. Similar workflows have been recently published by other institutions, which report 1.5 to 2 h for an offline adaptive workflow [20].

**Figure 7:**
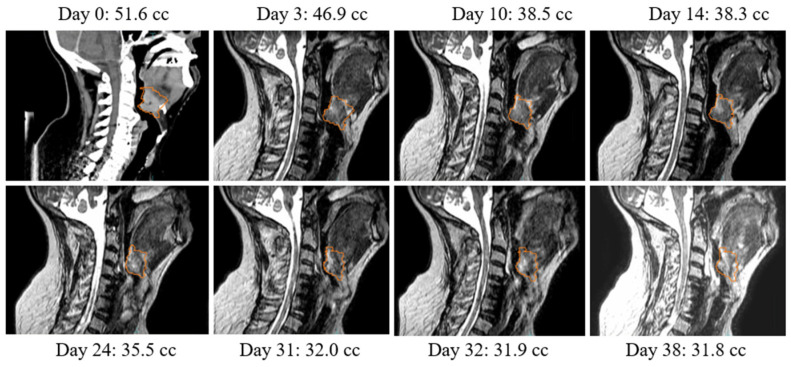
Regression of tumor volume over the course of treatment.

For the 15 patients treated for head and neck cancer, 27 offline adaptions have been performed and 8 online adaptions have been performed. One patient was adapted a total of 8 times offline to account for both tumor regression and variations of patient anatomy, shown in Figure 7. The tumor volume decreased by approximately 38% of the initial volume over the course of treatment. Four of the offline adaptions were performed for reasons other than tumor regression, such as patient weight loss or variations in patient positioning.

### 3.3. Extremities

Multiple extremity sites have been treated including patients with sarcomas of the thigh, foot, and hand. Patients with extremity targets selected for treatment using MRgART typically have lesions that are either easily visualized on daily MRI or the variations in daily position, swelling, and tumor regression must be accounted for with daily plan adaptions. Immobilization for these patients typically includes an indexed Vac-Lok and carefully considered positioning to ensure the extremity is as close to the center of the bore as possible. Such positioning maximized the useable field size and provided the best image quality.

Many of the extremities treated are shallow targets requiring superficial coverage, traditionally achieved using bolus. Not all bolus materials are visible during MRI imaging, and unexpected air gaps significantly reduce skin dose during delivery [21]. Clinically, an MR-safe, silicone bolus was commissioned, which is visible on both T1 and T2-weighted images (Klarity Medical Products, Heath, OH, USA) for MRgART to permit visualization and elimination of air gaps as required.

### 3.4. Brain

Brain treatments benefit from MRgART due to the excellent soft-tissue contrast and visualization of neurological structures utilizing different imaging sequences. Immobilization for brain treatments is performed using an acrylic baseplate similar to the one shown in Figure 6 (see discussion regarding treatment planning with a baseplate) indexable to the tabletop, which permits the use of thermoplastic masks and head rests. Simulation MRI scans include a T2 FLAIR and contrast-enhanced T1. Treatment for patients with GBMs typically involves delivering 4500 cGy to the edema clinical target volume (CTV) with a sequential boost to the gross tumor volume (GTV) identified on the contrast-enhanced T1 image.

**Figure 8:**
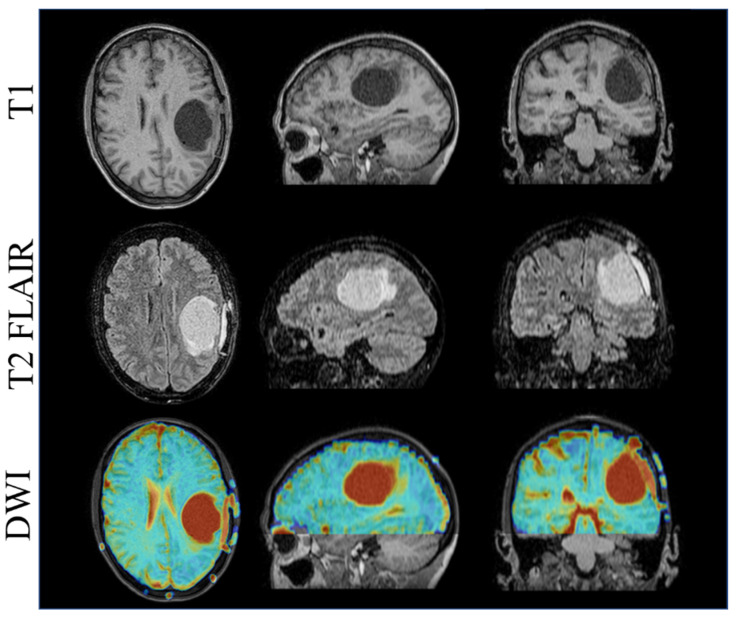
T1 (**Top**), T2 FLAIR (**Center**), and DWI (**Bottom**)-imaging 1.5T MR-linac.

Practically, this is accomplished by imaging with a T2 FLAIR pretreatment-imaging sequence for the initial course, allowing for edema visualization and adaption as the edema volume changes, followed by T1 imaging for the sequential boost to the high-dose target (Figure 8). Patients with targets near critical neurological structures such as the brainstem or spinal cord also benefit from the daily adaption of the contours to ensure dose limits are met for these critical serial organs. Care during planning is required to meet these dose constraints due to the coplanar-only geometry of the system. Diffusion-weighted imaging (DWI) is also acquired for patients receiving MRgRT brain treatments. Apparent diffusion coefficient (ADC)-quantitative maps are generated using b = 0 s and b = 1000 s EPI-based scans. These data are being processed in our department as part of a research effort to determine the clinical value in ADC changes over the course of treatment. The acquisition of DWI for each patient treatment adds no additional time since they are acquired during radiation-beam on.

## 4. Discussion

Due to the novel nature of adaptive therapy and MRgRT at our institution, the program is constantly improving and expanding. The speed of these developments across all facets of the treatment workflow warrant maintaining current knowledge and diligent attention to workflows. Second-check algorithms performing Monte Carlo calculations or incorporating asymmetric field shapes are being developed by multiple vendors in the field, providing more accurate validations of the TPS. Improvements from the vendor demonstrate potential to improve patient throughput and treatment workflows through AI-contouring tools, gated delivery, improved optimization algorithms, and the potential for helical treatments [22,23,24]. While helical treatments would permit the treatment of targets larger than the current field of view, the development of efficient and accurate AI-based contouring tools may reduce the required specialized personnel for online adaptive treatments and lead to more therapist-driven workflows.

While ATS workflows will likely remain a task of the medical physicist until these tools are further developed, the eventual goal of transitioning all ATP treatments to radiation-therapist-led workflow is attainable with the current tools and additional training. Additional roles have already been transitioned to the radiation therapists under the supervision of the medical physicist, including initial registration, second-check calculations, and data-transfer integrity. With more time and development of workflows, a complete transition and training is anticipated in the future.

In summary, modern MRgART adaptive radiotherapy programs require a team-based approach from physicists, physicians, dosimetrists, and therapists to ensure safe and effective daily treatments. This manuscript outlines several of the key aspects to treatment planning and delivery for a number of sites but is not meant to represent an exhaustive list. Many of the recommendations made are a result of weekly meetings with the MR-linac team and the associated discussions of individual cases. A methodical and thorough approach to introducing additional treatment sites or techniques should include similar discussions with a comprehensive group representing all aspects of the MRgART team. The additional complexities of working in the MR environment with adaptive radiotherapy present clinical challenges, but when addressed properly present large opportunity for improved patient care.

## Data Availability

No new data were created or analyzed in this study. Data sharing is not applicable to this article.
